# Dynamical modeling of drug effect using hybrid systems

**DOI:** 10.1186/1687-4153-2012-19

**Published:** 2012-12-26

**Authors:** Xiangfang Li, Lijun Qian, Edward R Dougherty

**Affiliations:** 1 Department of Electrical and Computer Engineering, Texas A&M University, College Station, TX 77843, USA; 2 Department of Electrical and Computer Engineering, Prairie View A&M University, Prairie View, TX 77446, USA; 3 Computational Biology Division, Translational Genomics Research Institution, Phoenix, AZ 85004, USA; 4 Department of Bioinformatics and Computational Biology, University of Texas M.D. Anderson Cancer Center, Houston, TX 77030, USA

**Keywords:** Drug effect, Hybrid systems, PK/PD, Gene regulatory network (GRN), Dosing regimens

## Abstract

Drug discovery today is a complex, expensive, and time-consuming process with high attrition rate. A more systematic approach is needed to combine innovative approaches in order to lead to more effective and efficient drug development. This article provides systematic mathematical analysis and dynamical modeling of drug effect under gene regulatory network contexts. A hybrid systems model, which merges together discrete and continuous dynamics into a single dynamical model, is proposed to study dynamics of the underlying regulatory network under drug perturbations. The major goal is to understand how the system changes when perturbed by drugs and give suggestions for better therapeutic interventions. A realistic periodic drug intake scenario is considered, drug pharmacokinetics and pharmacodynamics information being taken into account in the proposed hybrid systems model. Simulations are performed using MATLAB/SIMULINK to corroborate the analytical results.

## Introduction

The ultimate goal of drug therapy is to modulate the phenotypic behavior of cells by altering the behavior of the gene and protein components of the cell [1]. This approach is possible because the phenotypic behavior of the cell reflects the dynamics of the gene and protein-based regulatory network. When it comes to drug therapeutics and disease modeling, the major goal is to understand how the system changes when perturbed and how to modify the system to achieve a desired outcome. To understand and exploit the complicated mapping between genome and phenome, especially in the context of drug discovery, it is critical to evaluate the regulatory interactions between the genes and proteins that form the gene regulatory network (GRN). To date, the hope of the rapid translation of “genes to drugs” has foundered on the reality that disease biology is complex and drug development must be driven by insights into biological responses [2]. A systems approach is crucial for moving biology from a descriptive to a predictive science [3,4]. This calls for appropriate modeling to establish a functional understanding of disease–drug interaction, in order to better predict drug effects and make drug discovery a faster and more systematic process.

Pharmacokinetics (PK) is the study of *what the body does to the drug*, i.e., the absorption, distribution, metabolism, and excretion of the drug, and pharmacodynamics (PD) seeks to study *what the drug does to the body*. A salient challenge is to link a drug’s PK information with PD characteristics to provide a better understanding of the time course of drug effect (PK/PD) after drug administration [5]. Modeling and simulation tools are required to integrate PK and PD data and optimize drug regimens.

A salient problem is finding a dosing regimen of a drug candidate that is both efficacious and safe [6]. Traditionally, drugs have been administered on an experimental basis, but it is virtually impossible to optimize dosing regimens using strictly empirical methods, especially since different patients may respond differently to the same drug dosage [7]. Moreover, traditionally designing the dosing regimen to achieve some desired target goal such as relatively constant serum concentration may not be optimal because of underlying dynamic biological networks. For example, Shah et al. [8] demonstrate that BCR–ABL inhibitor dasatinib, which has greater potency and a short half-life, can achieve deep clinical remission in CML patients by achieving transient potent BCR–ABL inhibition, while traditionally approved tyrosine kinase inhibitors usually have prolonged half-lives that result in continuous target inhibition. A similar study of whether short pulses of higher dose or persistent dosing with lower doses has the most favorable outcomes has been carried out by Amin et al. [9] in the setup of inactivation of HER2–HER3 signaling. Finding an optimal dosing regimen based on the dynamics of biological systems and relevant PK/PD information is critically important.

System modeling is emerging as a valuable tool in therapeutics to address these challenges [3,10-12]. The process begins with building a quantitative model of a biological system. Consequences of particular perturbations, such as optimal dosing regimens, optimal drug targets, or combinational therapy, can be simulated in time courses using such models. In this study, we propose a hybrid systems model for GRNs and incorporate a drug’s PK and PD information by using a state-space approach. We first study drug effect assuming the drug target to be a gene or protein in the proposed drug perturbation model using dynamical system theory, considering the case of periodic drug intake and analytically deriving the conditions for the drug to be effective. We extend the analysis to the 2-gene case and then to the case of a network with multiple coupled genes and positive feedback loops. Simulations are performed using MATLAB/SIMULINK to supplement our analytical results.

## Model formulation

While discrete modeling leaves out many details, continuous modeling includes so many details that computational demands preclude their applications to many larger systems. Hybrid systems, which aim to merge ideas from both continuous and discrete modeling into one paradigm, are appealing for GRN modeling under drug perturbations because biological systems are naturally nonlinear, have highly varied regulatory requirements, and possess a wide range of control strategies for meeting their needs. While some simple, local, feedback control methods can provide sufficient regulation of many more-or-less continuous cellular processes, the regulation of discontinuous processes possessing the character of computational decision making requires more elaborate regulatory methods [13]. In particular, some genes display regulation in a thresholded switch-like manner [14].

Hybrid systems include a broad space of models and systems. Several hybrid systems models have been developed for biological networks. Some of these have been used to perform reachability analysis to elucidate biologically meaningful properties. For example, the Lac operon system has been well studied both experimentally and using continuous models [15,16]. A hybrid model and use of a reachability algorithm were validated by comparison with experimental data and continuous models [17]. Other biological hybrid systems analyzed in similar ways include the Delta-Notch decision process [18,19], GRNs of carbon starvation [20], and nutritional stress response [21] in *Escherichia coli*. As far as we know, the only hybrid systems modeling concerning treatment or drug effects is contained in our earlier work [22].

Gene regulation can be modeled by rate equations expressing the difference between rate of production and rate of degradation [23,24]. We adopt the general model

(1) $${\dot{x}}_{i}=f_{i}(x)-\gamma_{i}x_{i},$$

where *x*_*i*_≥0 corresponds to the concentrations of proteins encoded by genes in the network and can be interpreted as the gene expression level. *f*_*i*_(.) is a general nonlinear function and represents the rate of synthesis. It can be approximated by a sigmoidal function or a unit step function, and unit step function is used in this article. *γ*_*i*_*x*_*i*_ is the rate of degradation. To use hybrid systems and incorporate drug effect, we propose the following model for a GRN of *N* genes under drug perturbation:

(2) $$\begin{array}{l} {\dot{x}}_{i}=\overset{K_{i}}{\underset{k=1}{\sum }}\left\{\beta_{i}^{k}\left(\underset{j\in \Psi_{i}}{\prod }\Omega_{i}^{j}\right)\;\left[1-\left(\underset{j\in \Phi_{i}}{\prod }\Xi_{i}^{j}\right),\;\right],\;\right\}\\ \;-\gamma_{i}x_{i}+\beta_{i}^{u}\Omega_{i}^{u}-\gamma_{i}^{u}\Xi_{i}^{u}x_{i},\;\forall i=1,2,\ldots ,N, \end{array}$$

where the last two terms on the right-hand side of Equation (2) correspond to drug perturbation *u*. *β* > 0 and *γ* > 0 are synthesis and degradation rates, respectively. *K*_*i*_ ≥ 1 is an integer representing the number of activation/synthesis terms. $\Omega_{i}^{j}$ and $\Xi_{i}^{j}$ describe how other genes affect gene *i*. They are the functions of $s^{+}(x_{j},\theta_{j}^{t{+}_{i}})$ and $s^{-}(x_{j},\theta_{j}^{t{-}_{i}})$, where *s*^+^(.) is the unit step function, *s*^−^(.) = 1−*s*^+^(.), and the *θ* terms are the corresponding threshold values. For each gene *j*, the set of threshold values related to gene *i* is denoted by $T_{j}^{i}$, where *t*+_*i*_ and *t*−_*i*_ are indices of the threshold values, $0\leq \theta_{j}^{t{+}_{i}}\in T_{j}^{i}$, and $0\leq \theta_{j}^{t{-}_{i}}\in T_{j}^{i}$. *Ψ*_*i*_ and *Φ*_*i*_ represent the two sets of genes that affect the expression of gene *i* in different manners. Specifically, in this article, we consider

(3) $$\Omega_{i}^{j}=s^{+}(x_{j},\theta_{j}^{t{+}_{i}})s^{-}(x_{j},\theta_{j}^{t{-}_{i}}),$$

with $\Xi_{i}^{j}$ defined similarly. $\Omega_{i}^{j}$ and $\Xi_{i}^{j}$ may be set to 0 or 1, or different forms when appropriate threshold values are chosen. For example, $\Omega_{1}^{1}=s^{+}(x_{1},0^{-})s^{-}(x_{1},\infty )=1$ and $\Omega_{1}^{2}=s^{+}(x_{2},0^{-})s^{-}(x_{2},\theta_{1}^{2})=s^{-}(x_{2},\theta_{2}^{1})$. $\Omega_{i}^{u}$ and $\Xi_{i}^{u}$ describe how the drug *u* affect gene *i*. $\beta_{i}^{u}\geq 0$ and $\gamma_{i}^{u}\geq 0$ are the synthesis and degradation factors of the drug on gene *i*. $\beta_{i}^{u}\Omega_{i}^{u}$ and $-\gamma_{i}^{u}\Xi_{i}^{u}x_{i}$ are used when the drug is activating or repressing certain genes, respectively. Since most drugs are used to repress genes, only $-\gamma_{i}^{u}\Xi_{i}^{u}x_{i}$ is considered in the examples of this article. Note that *γ*^*u*^ is defined as a drug-effect factor, which is closely related to the drug pharmacology model discussed in the following section.

It should be kept in mind that the focus of this article is studying the effect of dosing, in particular, dosing regimens, on the expression of genes involved in a pathology by using hybrid systems theory. Whereas the simpler Equation (1) is widely accepted, it does not contain drug-effect terms. Equation (2) extends Equation (1) by including such terms. While the structure is intuitively reasonable and somewhat general, the actual details of the drug-effect terms are unknown. Finding the specific form of Equation (2) for a specific disease is a system identification problem, which is quite distinct from the analysis problem addressed in this article. We are addressing optimization of treatment intervention, given the system. The details of our analysis might change when the details of Equation (2) are clarified, but we expect that the hybrid systems approach taken in the article will go through with appropriate modifications in the mathematical details.

**Figure 1:**
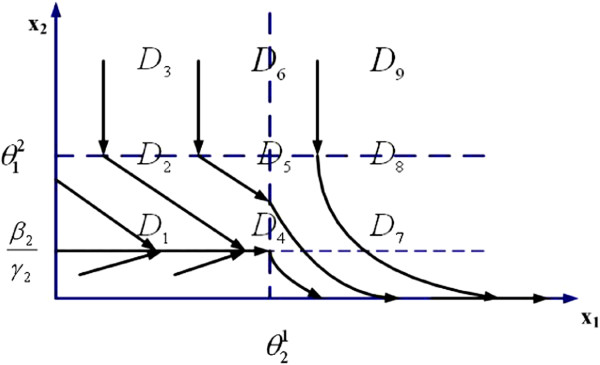
**State trajectory schematic of 2-gene example *without *drug intake.**

**Figure 2:**
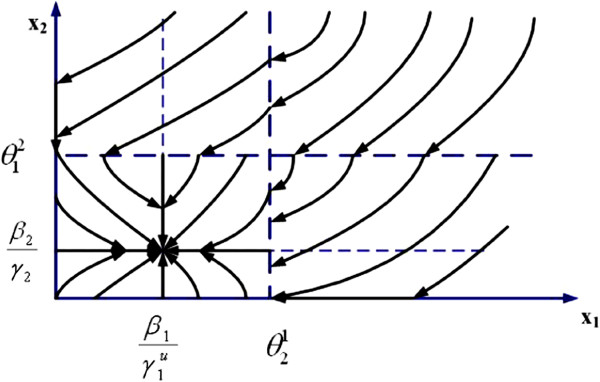
**State trajectory schematic of 2-gene example *with *drug intake.**

We consider a 2-gene example to illustrate the feasibility of using hybrid systems for modeling drug effect. Specifically, we assume that there are two interactive genes *x*_1_,*x*_2_ that repress each other, and *x*_1_ is a disease gene which loses its self-regulation. We also assume that a drug targets *x*_1_ by reducing its expression level and providing a negative feedback term $-\gamma_{1}^{u}x_{1}$. The resulting 2-gene network is given by

(4) $${\dot{x}}_{1}=\beta_{1}s^{-}(x_{2},\theta_{2}^{1})-\gamma_{1}^{u}x_{1}$$

(5) $${\dot{x}}_{2}=\beta_{2}s^{-}(x_{1},\theta_{1}^{2})-\gamma_{2}x_{2}$$

where *β*_1_, *β*_2_ are synthesis factors, *γ*_2_ is a degradation factor, and $\theta_{1}^{2},\;\theta_{2}^{1}$ are threshold values. $\gamma_{1}^{u}$ is a drug-effect factor. Using dynamical systems theory, the state-trajectory schematic diagrams of this 2-gene network without and with drug input are obtained and plotted in Figures 1 and 2, respectively. It is observed that without drug input, the gene expression level of *x*_1_ increases unbounded, while with proper drug input, $\beta_{1}/\gamma_{1}^{u}<\theta_{1}^{2}$, the system converges to a new steady state, $\left(\beta_{1}/\gamma_{1}^{u},\beta_{2}/\gamma_{2}\right)$.

**Figure 3:**
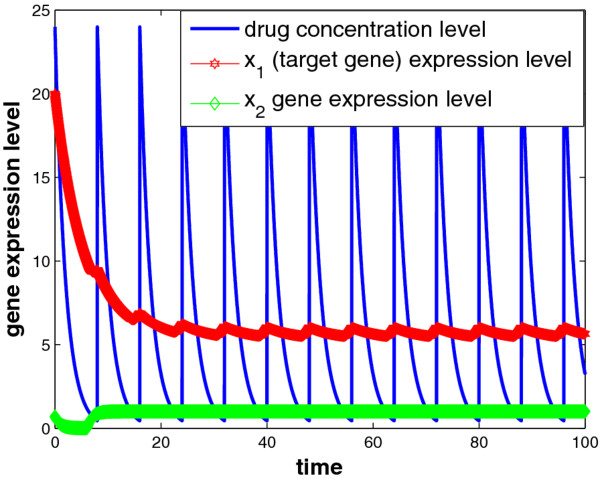
The state response under periodic drug intake.

**Figure 4:**
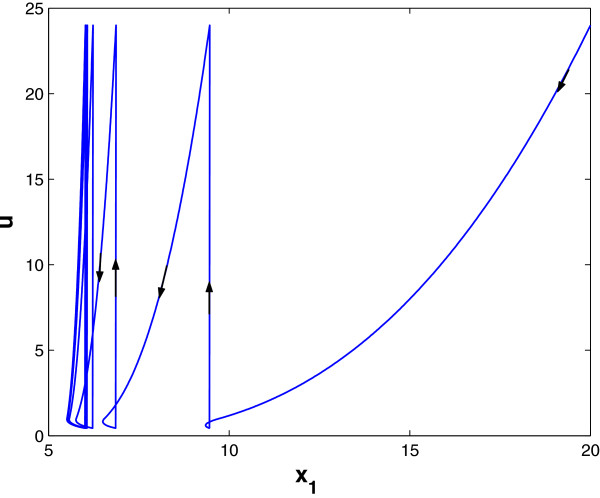
**The state-space trajectory under periodic drug intake.** Parameter setting of Figures 3 and 4: $x_{1}(0)=20,\;x_{2}(0)=0.7,\;\tau =8,u(\mathrm{k\tau})=24,\;q_{1}^{u}=0.21,\;\beta_{1}=\beta_{2}=1,\;\gamma_{2}=1,\;\theta_{1}^{2}=10,\;\theta_{2}^{1}=2,\lambda_{d}=0.5$.

**Figure 5:**
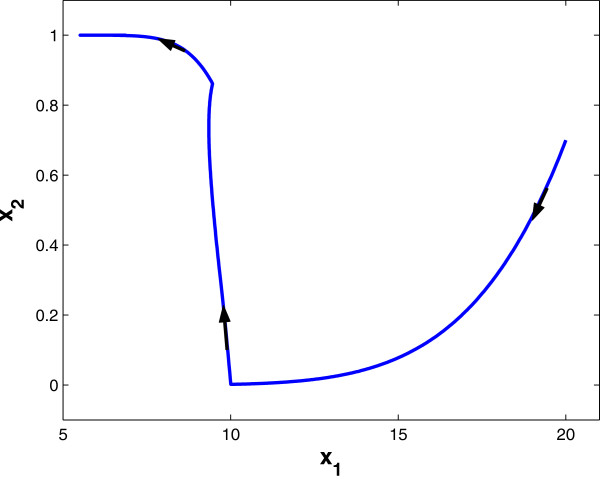
**The state-space trajectory *with *drug:**$\tau =8,u(\mathrm{k\tau})=24,q_{1}^{u}=0.21,\lambda_{d}=0.5$**.** The rest parameter settings are the same with Figures 3 and 4.

**Figure 6:**
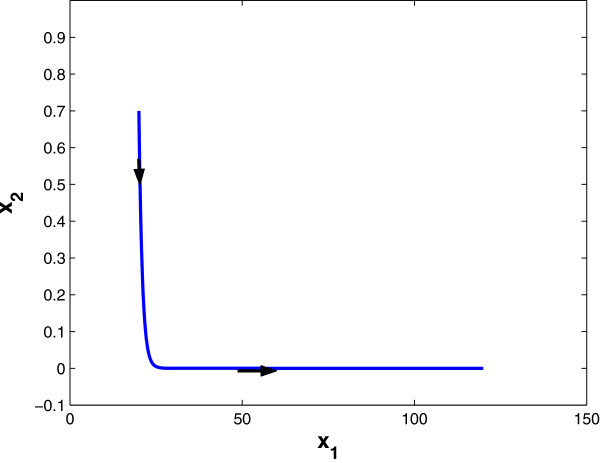
**The state-space trajectory *without *drug intake.** The rest parameter settings are the same with Figures 3 and 4.

We assume periodic drug intake and the drug concentration level in the effect-site follows exponential decay during each period *τ*_*i*_, i.e., $u_{i}(t)=\zeta_{i}e^{-\lambda_{d}(t-k\tau_{i})}$, where *kτ*_*i*_ ≤ *t* ≤ (*k* + 1)*τ*_*i*_ and *λ*_*d*_ is the degradation factor. The response of gene expression levels of the two genes under periodic drug intake is shown in Figure 3. The state-space trajectory of gene expression level of *x*_1_ vs. the drug concentration level *u* is given in Figure 4. A comparison of trajectory of the gene expression level *x*_1_ vs. *x*_2_ with and without drug are provided in Figures 5 and 6, respectively. It is observed that the drug is quite effective in bringing down the expression level of *x*_1_. The simulation study matches the theoretical analysis, as in Figures 1 and 2, that with proper drug intervention the system will converge to a new steady state, $x_{1}=\beta_{1}/\gamma_{1}^{u}$ and *x*_2_ = *β*_2_ / *γ*_2_ = 1, while *x*_2_ → 0 and *x*_1_ → *∞* without drug input.

## Pharmacology model

The basis of clinical pharmacology is the fact that the intensities of many pharmacological effects are functions of the amount of drug in the body and, more specifically, the concentration of drug at the effect-site [5]. Historically, PK and PD were considered as separate disciplines; however, the information provided by these disciplines is limited if regarded in isolation [25]. A drug-effect factor *γ*^*u*^ is included in our proposed model (Equation 2), which is related to drug’s PD characteristic (concentration–response) and its PK information (dose–concentration). In order to describe the time course of drug effect in response to different dosing regimens, the integrated PK/PD model is indispensable because it builds the bridge between these two classical disciplines of pharmacology [25]. Following each dosing regimen, instead of a two-dimensional PK and PD relationship, the proposed approach enables a description of a three-dimensional dose–concentration–effect relationship. Specifically, PK and PD are linked through *γ*^*u*^ by a state-space approach to facilitate the description and prediction of the time course of drug effects resulting from different drug administration regimens.

### Drug concentration–response curve: PD model

**Figure 7:**
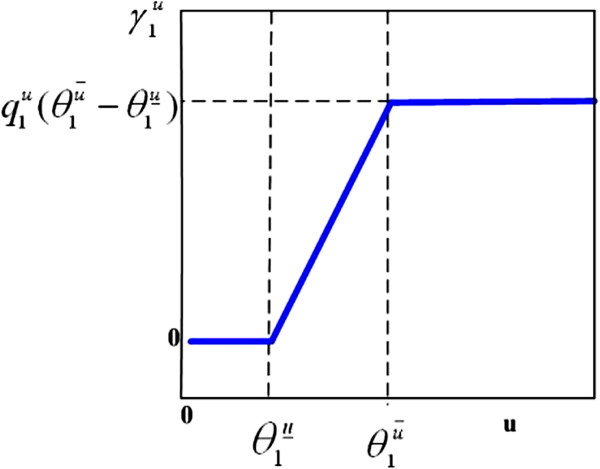
The PD model: concentration-response curve used in this study.

**Figure 8:**
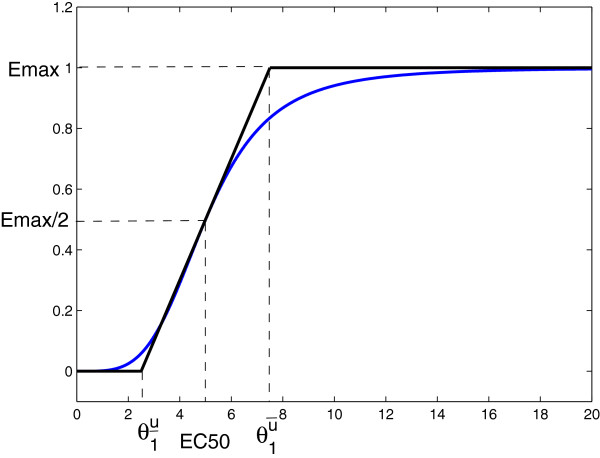
**Sigmoidal *E***_**max **_**model (*m=4*), and approximation by our PD model.**

In general, the magnitude of a pharmacological effect increases monotonically with increased dose, eventually reaching a plateau level where further increase in dose has little additional effect [6]. The classic and most commonly used concentration–response model is the Hill equation [26], also known as the sigmoidal *E*_max_ model [27] or logistic model [28]. The relationship between the concentration of the drug and its effect is most often nonlinear. In this study, we use hybrid systems to approximate PD curves. A common method is to replace certain slowly changing variables by their piecewise linear approximations (see Figure 7). For example, the PD model used in our study can approximate the popular sigmoidal *E*_max_ model (see Figure 8). The *E*_max_ model has the general form $E=\frac{E_{\max}C^{m}}{EC_{50}^{m}+C^{m}}$, where *E*_max_ is the maximum effect, *C* is the concentration, *EC*_50_ is the concentration necessary to produce 50% of *E*_max_, and *m* represents a sigmoidity factor or steepness of the curve.

We assume a threshold of concentration below which the drug candidate is ineffective, the minimum effective dose (MinED), and another threshold value, called maximum effective dose (MaxED), above which there is no clinically significant increase in pharmacological effect in this study. As an example, we use a linear curve to approximate the concentration–response curve between MinED and MaxED. It is assumed that the drug-effect coefficient $\gamma_{1}^{u}$ (the drug target is *x*_1_) is related to the concentration *u* through a sigmoid function and can be approximated by the curve shown in Figure 7. The corresponding relationship can be expressed as

(6) $$\gamma_{1}^{u}=\left\{\begin{array}{ll} \;0 & u<\theta_{1}^{\underset{‾}{u}}\\ {\;q}_{1}^{u}(u-\theta_{1}^{\underset{‾}{u}}) & \theta_{1}^{\underset{‾}{u}}\leq u\leq \theta_{1}^{\bar{u}}\\ {\;q}_{1}^{u}(\theta_{1}^{\bar{u}}-\theta_{1}^{\underset{‾}{u}}) & u>\theta_{1}^{\bar{u}} \end{array}\right),$$

where $q_{1}^{u}$ is the ratio between the drug-effect factor $\gamma_{1}^{u}$ and the effective drug concentration $u-\theta_{1}^{\underset{‾}{u}}$ in the linear range. This reflects the fact that the drug only starts to take effect when its concentration level is above a lower threshold $\theta_{1}^{\underset{‾}{u}}$ and its effect saturates when its concentration level exceeds an upper threshold $\theta_{1}^{\bar{u}}$. Note that the sigmoidal *E*_max_ model can be well approximated by the proposed PD model. By taking the derivative of *E* with respect to *C* and evaluating it at *EC*_50_, we obtain the slope as $q_{1}^{u}=\frac{mE_{\max}}{4EC_{50}}$. The upper and lower bounds should satisfy $q_{1}^{u}(\theta_{1}^{\bar{u}}-\theta_{1}^{\underset{‾}{u}})=E_{\max}$. An example of the sigmoidal *E*_max_ model when *m*=4 and our proposed PD model are plotted together in Figure 8, where the proposed model closely resembles the sigmoidal *E*_max_ model.

### Periodic drug intake: PK model

Drug concentration at the effect-site is critical for its pharmacological effect. Currently, plasma drug concentrations are markers that serve as surrogates for drug concentration at the effect-site for beneficial and adverse effects; however, markers not grounded on a sound theoretical foundation and therapeutic mechanism-based intervention can limit the usefulness of PK/PD modeling to drug development. For example, it has been demonstrated that the intracellular PK of a drug is quite different from plasma drug concentration [29,30]. As observed in the study by Kuh et al. [29], the intracellular concentration of a drug will exponentially increase as the drug is absorbed after each drug intake. The drug concentration may change very slowly (in our model, we approximate that as a flat curve) when the intracellular and extracellular drug concentration approach equilibrium. In time, drug concentration will exponentially decrease as the rate at which it is eliminated is more than the rate at which it enters the effect-site and, as a result, effects diminish.

**Figure 9:**
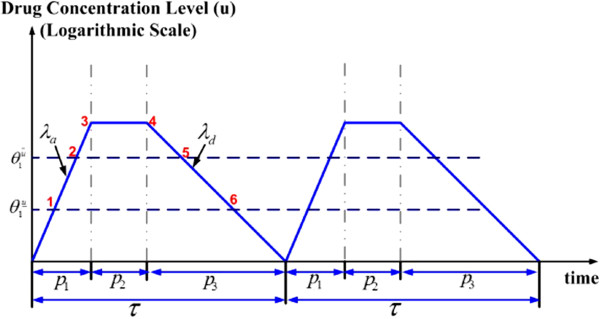
A general drug concentration-time profile.

Based on the study by Kuh et al. [29], a general model for drug concentration-time profile is given in Figure 9. Drug concentration is plotted on a logarithmic scale against time following each periodic drug intake. *λ*_*a*_ denotes the exponential increase quotient; *λ*_*d*_ is the exponential decrease quotient; *τ* is the interval between each drug intake; and *p*_1_, *p*_2_, and *p*_3_ denote the time stayed in the increase, equilibrium, and decrease stage, respectively. Different drugs work in different ways and the proposed model is general enough to cover various cases. Drug concentration may increase very quickly and, as a result, the increase stage may be neglected, or the equilibrium stage may be very short and can be ignored for simplicity. By adjusting the parameters in the proposed model, specific drug characteristics can be represented. In the case when the proposed model cannot approximate a drug’s PK profile, extensive simulations can be performed based on the drug’s actual PK profile. In this article, we consider a periodic drug intake scenario. Specifically, we are interested in investigating and comparing the following two potential scenarios: large dose with a longer interval versus small dose with a shorter interval.

## Mathematical analysis of drug effect

In this section, we study the time course of drug effect for different dosage and schedule arrangements where the drug is designed to repress a “target gene”. The case with a special PK profile (drug concentration only has exponential decay) was analytically studied in our previous work [22]. In this study, we extend the analysis considering a general PK profile given in Figure 9 and PD model given in Figure 8. Closed-form analytical solution is provided and simulations are performed to validate theoretical analysis. In later sections, we show that the same methodology can be applied to interactive genes, where not only will the drug affect the gene expression level, but the target gene is also coupled with other genes.

It is assumed that the disease gene has lost part of its self-regulation capacity and the dynamical equation of the expression level *x*_1_ is given by

(7) $${\dot{x}}_{1}=\beta_{1}-\gamma_{1}x_{1}.$$

There is a steady state *x*_1_ = *β*_1_ / *γ*_1_ in such a system, however, if the synthesis rate is much bigger than the self-degradation rate, *β*_1_ ≫ *γ*_1_, then the gene expression level will be too high. A drug is used as a control input to repress the target gene expression level. The corresponding dynamical equation after drug intake is changed to

(8) $${\dot{x}}_{1}=\beta_{1}-\gamma_{1}x_{1}-\gamma_{1}^{u}x_{1},$$

where $\gamma_{1}^{u}$ is the drug-effect factor defined in the previous section. After incorporating a drug’s PK/PD (Figures 7 and 9) into our proposed hybrid system model Equation (8), considering the scenario that the patient is taking the drug periodically, the resulting model is given by

(9) $$\begin{array}{l} \;{\dot{x}}_{1}\;=\beta_{1}-\gamma_{1}x_{1}-q_{1}^{u_{i}}(u_{i}-\theta_{1}^{{\underset{‾}{u}}_{i}})s^{+}(u_{i},\theta_{1}^{{\underset{‾}{u}}_{i}})s^{-}(u_{i},\theta_{1}^{{\bar{u}}_{i}})x_{1}\\ \;-q_{1}^{u_{i}}(\theta_{1}^{{\bar{u}}_{i}}-\theta_{1}^{{\underset{‾}{u}}_{i}})s^{+}(u_{i},\theta_{1}^{{\bar{u}}_{i}})x_{1},\\ \;u_{i}(t)\;=(e^{\lambda_{a}(t-k\tau_{i})}-1)s^{-}(t,k\tau_{i}+p_{1})\\ \;+\zeta_{i}s^{-}(t,k\tau_{i}+p_{1}+p_{2})s^{+}(t,k\tau_{i}+p_{1})\\ \;+\zeta_{i}e^{-\lambda_{d}(t-k\tau_{i}-p_{1}-p_{2})}s^{+}(t,k\tau_{i}+p_{1}\;+\;p_{2})s^{-}(t,(k+1)\tau_{i}), \end{array}$$

where for *kτ*_*i*_ ≤ *t* ≤ (*k*+1)*τ*_*i*_ and *i* = 1,2,… denoting the index of different dosing regimens, $q_{1}^{u_{i}}=q_{1}^{u}$, $\theta_{1}^{{\underset{‾}{u}}_{i}}=\theta_{1}^{\underset{‾}{u}}$, $\theta_{1}^{{\bar{u}}_{i}}=\theta_{1}^{\bar{u}},\;$ for any *i*, since we assume that the same drug is taken in different dosage and schedule settings. *ζ*_*i*_ is the highest concentration level reached after taking the drug.

### State-space analysis

**Figure 10:**
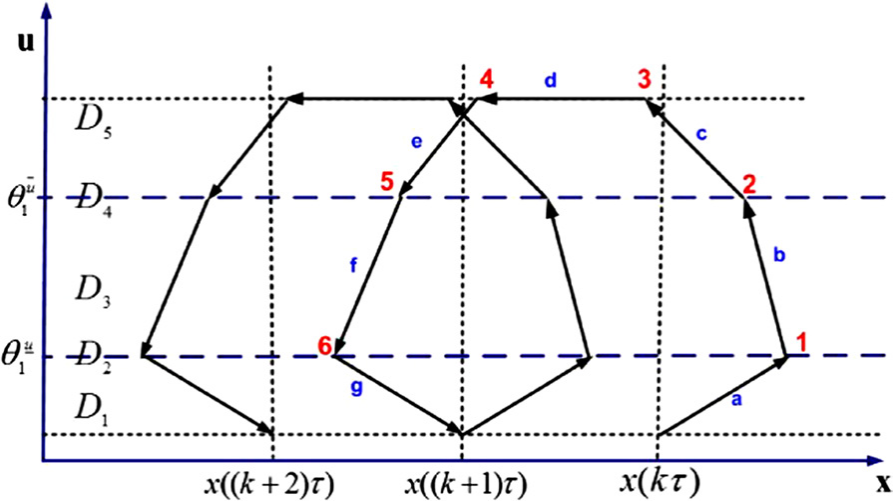
**The state trajectory schematic (target gene expression versus drug concentration) under PK profile (Figure **9**) assuming**$\text{dose}>\theta_{1}^{\bar{u}}$**.**

The state-space and a sample trajectory schematic of the state (target gene expression and drug concentration level) under periodic drug intake are shown in Figure 10. As is common in hybrid systems, there are both continuous quantitative changes and discrete transitions in our proposed model. The entire state space may be divided into different domains according to the value of discrete state. As shown in Figure 10, there are five domains in the state space, with *D*_1_,*D*_3_,*D*_5_ not being transient domains. The figure shows the case when the drug is effective and the drug dosage is large enough that *ζ*_*i*_ is higher than the upper threshold $\theta_{1}^{\bar{u}}$. The sample trajectory of the state corresponds to two periods of drug intake (numbers 1–6 corresponding to the junctions of the drug concentration and the boundaries of the domains, also marked in Figure 9). Another possible scenario is that the drug dosage is not large enough that *ζ*_*i*_ is between the upper threshold $\theta_{1}^{\bar{u}}$ and the lower threshold $\theta_{1}^{\underset{‾}{u}}$. The third scenario is the case that $\zeta_{i}<\theta_{1}^{\underset{‾}{u}}$ and the drug is not effective.

When the state transits in each period under periodic drug intake, it may pass through different domains (depending on the changes of drug concentration along time). During the transit time through domains *D*_5_ and *D*_3_, the gene expression level is pushed lower (to the left), while the driving strength will depend on the drug’s PD characteristic. During the transit time through *D*_1_, the expression level will rise (to the right), since the drug concentration is lower than $\theta_{1}^{\underset{‾}{u}}$. For the drug to be effective, the reduction of the expression level in *D*_5_ and *D*_3_ has to be larger than the increase of the expression level in *D*_1_. In summary, we should have *x*_1_((*k*+1)*τ*) < *x*_1_(*kτ*), so that after each treatment the expression level *x*_1_ will decrease.

### State trajectory analysis

We analyze the drug effect considering the scenario shown in Figure 10, where $\zeta_{i}>\theta_{1}^{\bar{u}}$. The same methodology can be applied to a simpler scenario where $\theta_{1}^{\underset{‾}{u}}<\zeta_{i}<\theta_{1}^{\bar{u}}$. We divide the state trajectory in a period *kτ*_*i*_ ≤ *t* ≤ (*k*+1)*τ*_*i*_ into stages a, b, c, d, e, f, and g as marked in Figure 10 and examine the drug effect stage-by-stage. The time notations used in the derivation are given by 

• *t*_1_: the traveling time from the initial state to the boundary between *D*_3_ and *D*_1_.

• *t*_2_: from initial state to boundary between *D*_5_ and *D*_3_.

• *t*_3_: from initial state to the end of stage c, *t*_3_ = *kτ*_*i*_+*p*_1_.

• *t*_4_: time at which the drug concentration starts to decrease, *t*_4_ = *kτ*_*i*_+*p*_1_+*p*_2_.

• *t*_5_: from the initial state to the end of stage e.

• *t*_6_: from the initial state to the end of stage f.

For *kτ*_*i*_ ≤ *t* ≤ (*k*+1)*τ*_*i*_, where *i* is the index for different dosing regimens, the corresponding equations and solutions for each stage are given by: 

• **Stage (*a*)** - *D*_1_ (*kτ*_*i*_ ≤ *t* ≤ *t*_1_):

(10) $$\begin{array}{llll} & & {\overset{\;˙}{\;x}}_{1}=\beta_{1}-\gamma_{1}x_{1},\;\;\Rightarrow & \\ & & {\;x}_{1}(t)=\frac{\beta_{1}}{\gamma_{1}}+\left[x_{1}(k\tau_{i})-\frac{\beta_{1}}{\gamma_{1}}\right]e^{-\gamma_{1}(t-k\tau_{i})}, & \\ & & {\;u}_{i}(t)=e^{\lambda_{a}(t-k\tau_{i})}-1. & \end{array}$$

• **Stage (*b*)** - *D*_3_ (*t*_1_≤* t *≤ *t*_2_):

(11) $$\begin{array}{l} {\dot{x}}_{1}=\beta_{1}-[\gamma_{1}+q_{1}^{u}(u-\theta_{1}^{\underset{‾}{u}})]x_{1}\;\;\Rightarrow \\ x_{1}(t)=\frac{x_{1}(t_{1})A(t_{1})}{A(t)}\\ \;+\frac{1}{A(t)}\overset{t}{\underset{t_{1}}{\int }}\beta_{1}e^{-[(q_{1}^{u}(1+\theta_{1}^{\underset{‾}{u}})-\gamma_{1})\sigma -\frac{q_{1}^{u}}{\lambda_{a}}e^{\lambda_{a}(\sigma -k\tau_{i})}]}\mathrm{d\sigma},\\ A(t)=e^{-[(q_{1}^{u}(1+\theta_{1}^{\underset{‾}{u}})-\gamma_{1})t-\frac{q_{1}^{u}}{\lambda_{a}}e^{\lambda_{a}(t-k\tau_{i})}]},\\ u_{i}(t)=e^{\lambda_{a}(t-k\tau_{i})}-1. \end{array}$$

• **Stage (*c*)** - *D*_5_ (*t*_2 _ ≤ *t* ≤ *t*_3_ = *kτ*_*i*_+*p*_1_):

(12) $$\begin{array}{l} {\dot{x}}_{1}=\beta_{1}-[\gamma_{1}+q_{1}^{u}(\theta_{1}^{\bar{u}}-\theta_{1}^{\underset{‾}{u}})]x_{1}\;\;\Rightarrow \\ x_{1}(t)=\frac{\beta_{1}}{\gamma_{1}+q_{1}^{u}(\theta_{1}^{\bar{u}}-\theta_{1}^{\bar{u}}-\theta_{1}^{\underset{‾}{u}})}\\ \;+\left[x_{1}(t_{2})-\frac{\beta_{1}}{\gamma_{1}+q_{1}^{u}(\theta_{1}^{\underset{‾}{u}})}\right]\\ \;\times e^{-[\gamma_{1}+q_{1}^{u}(\theta_{1}^{\bar{u}}-\theta_{1}^{\underset{‾}{u}})](t-t_{2})},\\ u_{i}(t)=e^{\lambda_{a}(t-k\tau_{i})}-1 \end{array}$$

• **Stage (*d*)** - *D*_5_ (*t*_3_ ≤ *t* ≤ *t*_4_ = *kτ*_*i*_ + *p*_1_ + *p*_2_):

(13) $$\begin{array}{l} {\dot{x}}_{1}=\beta_{1}-[\gamma_{1}+q_{1}^{u}(\theta_{1}^{\bar{u}}-\theta_{1}^{\underset{‾}{u}})]x_{1}\;\;\Rightarrow \\ x_{1}(t)=\frac{\beta_{1}}{\gamma_{1}+q_{1}^{u}(\theta_{1}^{\bar{u}}-\theta_{1}^{\underset{‾}{u}})}\\ \;+\left[x_{1}(t_{3})-\frac{\beta_{1}}{\gamma_{1}+q_{1}^{u}(\theta_{1}^{\bar{u}}-\theta_{1}^{\underset{‾}{u}})}\right]\\ \;\times e^{-[\gamma_{1}+q_{1}^{u}(\theta_{1}^{\bar{u}}-\theta_{1}^{\underset{‾}{u}})](t-t_{3})},\\ u_{i}(t)=u_{i}^{\max}=\zeta_{i} \end{array}$$

• **Stage (*e*)** - *D*_5_ (*t*_4_ ≤ *t* ≤ *t*_5_):

(14) $$\begin{array}{l} {\dot{x}}_{1}=\beta_{1}-[\gamma_{1}+q_{1}^{u}(\theta_{1}^{\bar{u}}-\theta_{1}^{\underset{‾}{u}})]x_{1}\;\;\Rightarrow \\ x_{1}(t)=\frac{\beta_{1}}{\gamma_{1}+q_{1}^{u}(\theta_{1}^{\bar{u}}-\theta_{1}^{\underset{‾}{u}})}\\ \;+\left[x_{1}(t_{4})-\frac{\beta_{1}}{\gamma_{1}+q_{1}^{u}(\theta_{1}^{\bar{u}}-\theta_{1}^{\underset{‾}{u}})}\right]\\ \;\times e^{-[\gamma_{1}+q_{1}^{u}(\theta_{1}^{\bar{u}}-\theta_{1}^{\underset{‾}{u}})](t-t_{4})},\\ u_{i}(t)=\zeta_{i}e^{-\lambda_{d}(t-t_{4})}. \end{array}$$

• **Stage (*f*)** - *D*_3_ (*t*_5_ ≤ *t* ≤ *t*_6_):

(15) $$\begin{array}{l} {\dot{x}}_{1}=\beta_{1}-[\gamma_{1}+q_{1}^{u}(u-\theta_{1}^{\underset{‾}{u}})]x_{1}\;\;\Rightarrow \\ x_{1}(t)=\left[x_{1}(t_{5})e^{-[\frac{q_{1}^{u}}{\lambda_{d}}\theta_{1}^{\bar{u}}+(q_{1}^{u}\theta_{1}^{\underset{‾}{u}}-\gamma_{1})t_{5}]}\right)\\ \;\left(+\overset{t}{\underset{t_{5}}{\int }}\beta_{1}e^{-[\frac{q_{1}^{u}}{\lambda_{d}}\theta_{1}^{\bar{u}}e^{-\lambda_{d}(\sigma -t_{5})}+(q_{1}^{u}\theta_{1}^{\underset{‾}{u}}-\gamma_{1})\sigma ]}\mathrm{d\sigma}\right]\\ \;\times e^{\left[\frac{q_{1}^{u}}{\lambda_{d}}\theta_{1}^{\bar{u}}e^{-\lambda_{d}(t-t_{5})}+(q_{1}^{u}\theta_{1}^{\underset{‾}{u}}-\gamma_{1})t\right]},\\ u_{i}(t)=\theta_{1}^{\bar{u}}e^{-\lambda_{d}(t-t_{5})}. \end{array}$$

• **Stage (*g*)** - *D*_1_ (*t*_6_ ≤ *t* ≤ (*k*+1)*τ*_*i*_):

(16) $$\begin{array}{lc} \;{\dot{x}}_{1}=\beta_{1}-\gamma_{1}x_{1}\;\;\Rightarrow \\ x_{1}(t)=\frac{\beta_{1}}{\gamma_{1}}+\left[x_{1}(t_{6})-\frac{\beta_{1}}{\gamma_{1}}\right]e^{-\gamma_{1}(t-t_{6})},\\ u_{i}(t)=\theta_{1}^{\underset{‾}{u}}e^{-\lambda_{d}(t-t_{6})}. & \end{array}$$

We can deduce the necessary and sufficient condition for the effectiveness of the drug by expressing the inequality *x*_1_((*k*+1)*τ*) < *x*_1_(*kτ*) in terms of dosing period *τ* and unit dose, assuming the dosage is proportional to the maximum drug concentration *ζ*_*i*_ reached after taking the drug. When the initial conditions are *x*_1_ = *x*_1_(*kτ*_*i*_), the equations governing the state trajectory from time *kτ*_*i*_ to time (*k*+1)*τ*_*i*_ are given by

(17) $$x_{1}(t_{1})=\frac{\beta_{1}}{\gamma_{1}}+\left[x_{1}(k\tau_{i})-\frac{\beta_{1}}{\gamma_{1}}\right]e^{-\gamma_{1}(t_{1}-k\tau_{i})},$$

(18) $$\begin{array}{lll} \;x_{1}(t_{2}) & \;= & \frac{x_{1}(t_{1})A(t_{1})}{A(t_{2})}+\frac{1}{A(t_{2})}\\ & & \times \overset{t_{2}}{\underset{t_{1}}{\int }}\beta_{1}e^{-[(q_{1}^{u}(1+\theta_{1}^{\underset{‾}{u}})-\gamma_{1})\sigma -\frac{q_{1}^{u}}{\lambda_{a}}e^{\lambda_{a}(\sigma -k\tau_{i})}]}\mathrm{d\sigma},\\ \;A(t_{2}) & \;= & \;e^{-[(q_{1}^{u}(1+\theta_{1}^{\underset{‾}{u}})-\gamma_{1})t_{2}-\frac{q_{1}^{u}}{\lambda_{a}}e^{\lambda_{a}(t_{2}-c)}]}, \end{array}$$

(19) $$\begin{array}{ccc} \;x_{1}(t_{5}) & \;= & \frac{\beta_{1}}{\gamma_{1}+q_{1}^{u}(\theta_{1}^{\bar{u}}-\theta_{1}^{\underset{‾}{u}})}\\ & & +\left[x_{1}(t_{2})-\frac{\beta_{1}}{\gamma_{1}+q_{1}^{u}(\theta_{1}^{\bar{u}}-\theta_{1}^{\underset{‾}{u}})}\right]\\ & & e^{-[\gamma_{1}+q_{1}^{u}(\theta_{1}^{\bar{u}}-\theta_{1}^{\underset{‾}{u}})](t_{5}-t_{2})}, \end{array}$$

(20) $$\begin{array}{ccc} \;x_{1}(t_{6}) & \;= & \left[x_{1}(t_{5})e^{-[\frac{q_{1}^{u}}{\lambda_{d}}\theta_{1}^{\bar{u}}+(q_{1}^{u}\theta_{1}^{\underset{‾}{u}}-\gamma_{1})t_{5}]}\right)\\ & & \left(+\overset{t_{6}}{\underset{t_{5}}{\int }}\beta_{1}e^{-[\frac{q_{1}^{u}}{\lambda_{d}}\theta_{1}^{\bar{u}}e^{-\lambda_{d}(\sigma -t_{5})}+(q_{1}^{u}\theta_{1}^{\underset{‾}{u}}-\gamma_{1})\sigma ]}\mathrm{d\sigma}\right]\\ & & e^{\left[\frac{q_{1}^{u}}{\lambda_{d}}\theta_{1}^{\bar{u}}e^{-\lambda_{d}(t_{6}-t_{5})}+(q_{1}^{u}\theta_{1}^{\underset{‾}{u}}-\gamma_{1})t_{6}\right]}, \end{array}$$

(21) $$\begin{array}{c} x_{1}((k+1)\tau_{i})=\frac{\beta_{1}}{\gamma_{1}}+\left[x_{1}(t_{6})-\frac{\beta_{1}}{\gamma_{1}}\right]e^{-\gamma_{1}((k+1)\tau_{i}-t_{6})}, \end{array}$$

(22) $$t_{1}=k\tau_{i}+\frac{1}{\lambda_{a}}\ln(1+\theta_{1}^{\underset{‾}{u}}),$$

(23) $$t_{2}=k\tau_{i}+\frac{1}{\lambda_{a}}\ln(1+\theta_{1}^{\bar{u}}),$$

(24) $$p_{1}=\frac{1}{\lambda_{a}}\text{ln}(1+\zeta_{i}),$$

(25) $$t_{5}=k\tau_{i}+p_{1}+p_{2}+\frac{1}{\lambda_{d}}\ln\left(\frac{\zeta_{i}}{\theta_{1}^{\bar{u}}}\right),$$

(26) $$t_{6}=t_{5}-\frac{1}{\lambda_{d}}\ln\left(\frac{\theta_{1}^{\underset{‾}{u}}}{\theta_{1}^{\bar{u}}}\right),$$

For the drug to be effective, we need the disease gene expression level to decrease following each period of drug intake. Hence, we can express *x*_1_((*k*+1)*τ*) < *x*_1_(*kτ*) in terms of dosage and frequency schedule and derive the region where the drug is effective using the above listed equations.

### Results and analysis

**Figure 11:**
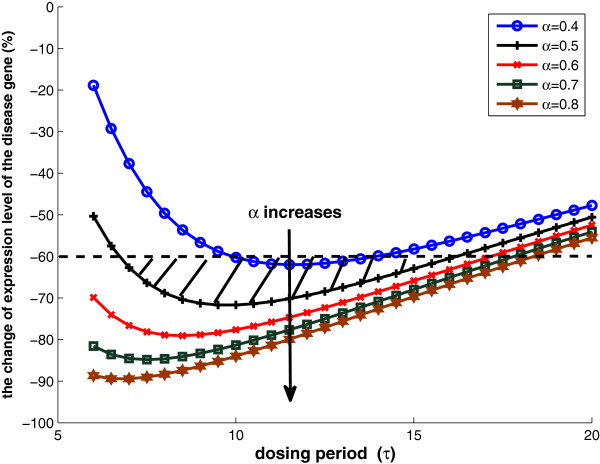
**The percentage change of the disease gene expression versus the period of drug intake *τ *for *α *= 0.4,0.5,0.6,0.7,0.8, respectively.** The change must be significant (say at least 60%) and *α* must be smaller than the acceptable toxic level. Parameters used: $q_{1}^{u}=0.1,\beta_{1}=1,\;\gamma_{1}=0.04,\;\theta_{1}^{\bar{u}}=10,\;\theta_{1}^{\underset{‾}{u}}=2,\;x(0)=20;\;p_{1}=1;\;p_{2}=5;\;\lambda_{d}=0.3$.

Based on the theoretical analysis in previous two sections, we demonstrate that the drug efficacy depends on total drug intake, different dosages, and frequencies. The density of drug intake is defined as $\alpha =\frac{\text{Dose}1}{\tau_{1}}=\frac{\text{Dose}2}{\tau_{2}}$. It is proportional to the total drug intake, and hence, related to the drug toxicity level in practice. First, we demonstrate the effect of total drug intake (equivalently, *α*) towards drug efficacy. For each fixed total drug intake, we plot the target gene expression reduction based on Equations 17 to 26 for different dosing period *τ* as a curve in Figure 11. It is observed that the curve is “U” shaped and there exist “sweet spot” for certain dosages and schedules given a fixed *α*. If we define *drug efficacy region* (DER) as the drug drives down the target gene expression by more than a desired percentage (say 60% in this case), it is demonstrated that the DER is related to the total drug intake, dosing period *τ* and dosage. DER is marked by the shaded area in Figure 11 for the case that *α* ≤ 0.5. It is also observed that when *α* gets bigger, which indicating more toxicity, DER is getting bigger accordingly. We would like to emphasize that toxicity is one of the primary causes for drug attrition and long development cycle times [31]. If a drug’s toxicity profile is available, for example, the maximum dosage and maximum exposure (*α*), we can find a good compromise between toxicity and drug efficacy based on such study, and determine the “sweet spot” (a good dosage and schedule balance) given the obtained *α*, and hence provide valuable suggestions of the dosing regimens to the clinical study.

**Figure 12:**
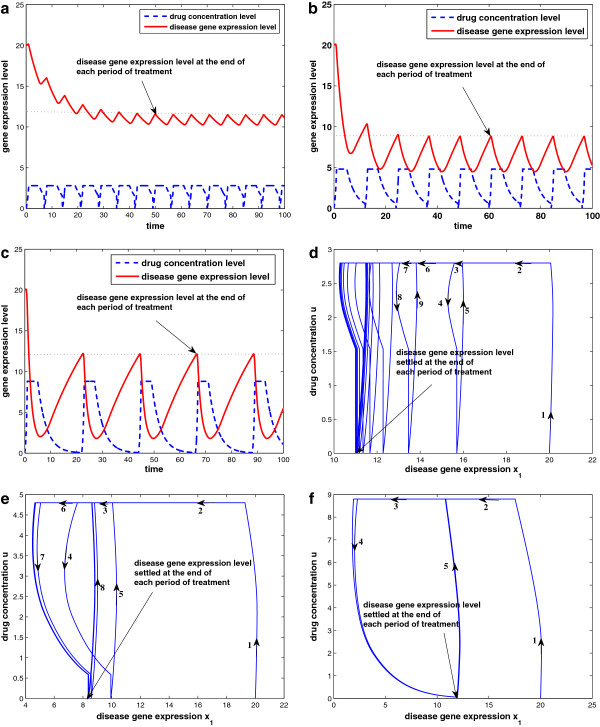
**Drug response at three different schedules but same drug intake: (a–c) the state response at τ = 7,12,22, respectively; (d–f) the state space trajectory at τ = 7,12,22, respectively.** Other parameters are $q_{1}^{u}=0.1,\;\beta_{1}=1,\;\gamma_{1}=0.04,\;\theta_{1}^{\bar{u}}=10,\;\theta_{1}^{\underset{‾}{u}}=2,\;x(0)=20;p_{1}=1;\;p_{2}=5;\;\lambda_{d}=0.3$.

Second, we test the analytical results via numerical simulation using MATLAB/SIMULINK. Given a fixed total drug intake, or equivalently, a fixed density of drug intake (*α* = 0.4), three typical scenarios are studied by simulation: small frequent drug intake with *τ* = 7 and *dose* = 2.8; big infrequent drug intake with *τ* = 22 and *dose* = 8.8; and intermediate dosage and frequency with *τ* = 12 and *dose* = 4.8. The results are shown in Figure 12a–f, with the first row corresponding to the state responses and the second row corresponding to the state space trajectory. Although the three cases have the same total drug intake and initial condition (initial gene expression level *x*(0) = 20), the drug efficacy is different. In the small frequent intake case, the dosage is small and the drug concentration is mostly changing between domains *D*_3_ and *D*_1_. Figure 12d shows that the state-space trajectory settles in a small limit cycle and disease gene expression level settles at 11.5 at the end of each period of treatment. On the other hand, the big infrequent drug intake case results in a big limit cycle as in Figure 12f. Although the dosage is high, the long period between dosages means that the period stayed in *D*_1_ is getting longer (where drug concentration is below $\theta_{1}^{\underset{‾}{u}}$, hence not effective), and disease gene expression level settles at 12.1 at the end of each period of treatment. As a comparison, the drug effect for the case with intermediate dosage and frequency shown in Figure 12b,e is superior to the other two cases. Disease gene expression level settles at 8.8 at the end of each treatment period. If we check the curve in Figure 11 with *α* = 0.4, the intermediate dosage case with *τ* = 12 is located near the bottom of the “U” shape. Lastly, we observe that all state-space trajectories follow the state trajectory schematic in Figure 10, as predicted by the analytical results.

## Analysis of 2-gene networks

**Figure 13:**
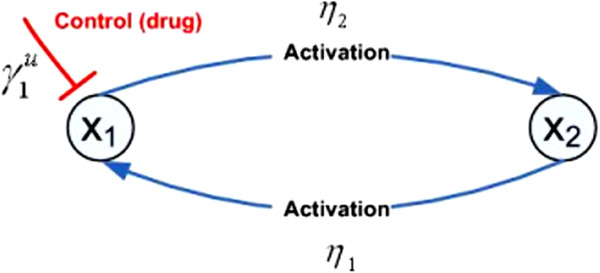
A 2-gene network with positive feedback loop and drug input.

We extend the theoretical analysis to 2-gene networks and show that the same framework applies to the modeling and analysis of drug effect in more complex gene networks. We assume that *x*_1_ is the target gene, there exists a positive feedback loop between *x*_1_ and another gene *x*_2_, and that a drug targets *x*_1_ by reducing its expression level and providing a negative feedback term $-\gamma_{1}^{u}x_{1}$. The resulting 2-gene network is shown in Figure 13 and is given by

(27) $$\begin{array}{lc} {\dot{x}}_{1}=\beta_{1}+\eta_{1}x_{2}-\gamma_{1}x_{1}-\gamma_{1}^{u}x_{1} & \\ {\dot{x}}_{2}=\beta_{2}+\eta_{2}x_{1}-\gamma_{2}x_{2} & \end{array}$$

where *β*_1_, *β*_2_ are synthesis factors, *γ*_1_ and *γ*_2_ are degradation factors, and $\gamma_{1}^{u}$ is a drug-effect factor. *η*_1_ > 0 and *η*_2_ > 0 are the parameters of the positive feedback loop between the two genes. The 2-gene network under drug perturbation model, Equation (27), can be rewritten as a second-order ODE:

(28) $$\;{\ddot{x}}_{1}+(\gamma_{1}+\gamma_{1}^{u}+\gamma_{2}){\dot{x}}_{1}+((\gamma_{1}+\gamma_{1}^{u})\gamma_{2}-\eta_{1}\eta_{2})x_{1}=\beta_{1}\gamma_{2}+\beta_{2}\eta_{1}$$

The solution of this equation is given by

(29) $$x_{1}(t)=\left\{\begin{array}{cc} k_{1}e^{\lambda_{1}t}+k_{2}e^{\lambda_{2}t}\; & \lambda_{1}\neq \lambda_{2}\\ k_{1}e^{\lambda_{1}t}+k_{2}te^{\lambda_{1}t}\; & \lambda_{1}=\lambda_{2} \end{array}\right),$$

where *λ*_1_ and *λ*_2_ are the two eigenvalues of Equation 28 and *k*_1_ and *k*_2_ are parameters depending on the initial conditions. Letting $a=\gamma_{1}+\gamma_{1}^{u}+\gamma_{2}$, $b=(\gamma_{1}+\gamma_{1}^{u})\gamma_{2}-\eta_{1}\eta_{2}$ and *d* = *β*_1_*γ*_2_ + *β*_2_*η*_1_, the two eigenvalues are given by $\lambda_{1,2}=\frac{-a\pm \sqrt{a^{2}-4b}}{2}$. It is easy to verify that $a^{2}-4b={\left(\gamma_{1}+\gamma_{1}^{u}-\gamma_{2}\right)}^{2}+4\eta_{1}\eta_{2}>0$. Since *a*^2^−4*b* > 0, we conclude that *λ*_1_ ≠ *λ*_2_ and both eigenvalues are real. Furthermore, one of the eigenvalues, *λ*_2_, is always negative since $\lambda_{2}=\frac{-a-\sqrt{a^{2}-4b}}{2}<0$. The sign of *λ*_1_ will be determined by the sign of *b*:

(30) $$\begin{array}{cc} \lambda_{1}<0,\; & \text{if}\;\;b>0\\ \lambda_{1}=0,\; & \text{if}\;\;b=0\\ \lambda_{1}>0,\; & \text{if}\;\;b<0 \end{array}$$

In other words,

(31) $$\begin{array}{cc} \lambda_{1}<0,\;\lambda_{2}<0\; & \text{if}\;(\gamma_{1}+\gamma_{1}^{u})\gamma_{2}>\eta_{1}\eta_{2}\\ \lambda_{1}=0,\;\lambda_{2}<0\; & \text{if}\;(\gamma_{1}+\gamma_{1}^{u})\gamma_{2}=\eta_{1}\eta_{2}\\ \lambda_{1}>0,\;\lambda_{2}<0\; & \text{if}\;(\gamma_{1}+\gamma_{1}^{u})\gamma_{2}<\eta_{1}\eta_{2} \end{array}.$$

The above equation has an important biological interpretation: when the degradation of *x*_1_ due to the strength of the drug is faster than the increase of *x*_1_ due to the positive feedback loop, both eigenvalues are negative, the system is stable and *x*_1_ will experience exponential decay; on the other hand, if the effect of the positive feedback loop is dominant, then one of the eigenvalues will be positive and *x*_1_ will increase exponentially.

Given initial condition *x*_1_(*t*_0_) and ${\dot{x}}_{1}(t_{0})$, then for the case *λ*_1_ ≠ *λ*_2_, we have

(32) $$k_{1}=\frac{e^{-\lambda_{1}t_{0}}}{\lambda_{1}-\lambda_{2}}(d\lambda_{2}/b+{\dot{x}}_{1}(t_{0})-\lambda_{2}x_{1}(t_{0}))$$

(33) $$k_{2}=\frac{e^{-\lambda_{2}t_{0}}}{\lambda_{2}-\lambda_{1}}(d\lambda_{1}/b+{\dot{x}}_{1}(t_{0})-\lambda_{1}x_{1}(t_{0}))$$

Now with the baseline analysis of the second-order system, we provide detailed state trajectory analysis by taking into account the practical form of PK/PD ($\gamma_{1}^{u}$) when the drug is taken periodically.

### State trajectory analysis

We analyze the drug-effect following the same framework given in the subsection “State trajectory analysis” under the main section “Mathematical analysis of drug effect”. For *kτ*_*i*_ ≤ *t* ≤ (*k*+1)*τ*_*i*_, *i* = 1,2,…, the corresponding equations and solutions for each stages are given as follows: 

• **Stage (*a*)** - *D*_1_ (*kτ*_*i*_ ≤ *t* ≤ *t*_1_):

(34) $$\begin{array}{cccc} {\dot{x}}_{1} & = & \beta_{1}+\eta_{1}x_{2}-\gamma_{1}x_{1} & \\ {\dot{x}}_{2} & = & \beta_{2}+\eta_{2}x_{1}-\gamma_{2}x_{2}\\ u_{i}(t) & = & e^{\lambda_{a}(t-k\tau_{i})}-1. & \end{array}$$

• The solution of *x*_1_(*t*) is given by Equation (29), with *k*_1_ and *k*_2_ given by Equations (32) and (33), and *t*_0_ = *kτ*_*i*_.

• **Stage (*b*)** - *D*_3_(*t*_1_ ≤ *t* ≤ *t*_2_):

(35) $$\begin{array}{cccc} \;{\dot{x}}_{1} & = & \beta_{1}+\eta_{1}x_{2}-\gamma_{1}x_{1}-\gamma_{1}^{u}x_{1}\\ \;{\dot{x}}_{2} & = & \beta_{2}+\eta_{2}x_{1}-\gamma_{2}x_{2}\\ \;\Rightarrow & & \\ \;{\ddot{x}}_{1}+a{\dot{x}}_{1}+bx_{1} & = & d\\ \;u_{i}(t) & = & e^{\lambda_{a}(t-k\tau_{i})}-1 & \end{array}$$

• where *a, b, d* are defined as before. When incorporating the practical form of $\gamma_{1}^{u}=q_{1}^{u}(u-\theta_{1}^{\underset{‾}{u}})$ and $u=e^{\lambda_{a}(t-k\tau_{i})}-1$, the above second-order ODE has no closed-form solution. In this case, the solution can be obtained numerically.

• **Stage (*c*)** - *D*_5_ (*t*_2_ ≤ *t* ≤ *t*_3_ = *kτ*_*i*_ + *p*_1_): The set of equations are the same as in Stage (*b*) except that $\gamma_{1}^{u}=q_{1}^{u}(\theta_{1}^{\bar{u}}-\theta_{1}^{\underset{‾}{u}})$. Since $\gamma_{1}^{u}$ does *not* depend on $u=e^{\lambda_{a}(t-k\tau_{i})}-1$ explicitly, *x*_1_ has a closed-form solution given by Equation (29).

• **Stage (*d*)** - *D*_5_ (*t*_3_ ≤ *t* ≤ *t*_4_ = *kτ*_*i*_ + *p*_1_ + *p*_2_): The solution of *x*_1_ is the same as that in Stage (*c*) except the start and end times, and the equation of *u*, which is $u_{i}(t)=u_{i}^{\max}=\zeta_{i}$ in this stage.

• **Stage (*e*)** - *D*_5_ (*t*_4_ ≤ *t* ≤ *t*_5_): The solution of *x*_1_ is the same as in Stage (*c*) except the start and end times, and the equation of *u*, which now is $u_{i}(t)=\zeta_{i}e^{-\lambda_{d}(t-t_{4})}$.

• **Stage (*f*)** - *D*_3_ (*t*_5_ ≤ *t* ≤ *t*_6_): The solution of *x*_1_ is the same as in Stage (*b*) except the start and end times, and the equation of *u*, which now is $u_{i}(t)=\theta_{1}^{\bar{u}}e^{-\lambda_{d}(t-t_{5})}$.

• **Stage (*g*)** - *D*_1_ (*t*_6_ ≤ *t* ≤ (*k*+1)*τ*_*i*_): The solution of *x*_1_ is the same as in Stage (*a*) except the start and end times, and the equation of *u*, which now is $u_{i}(t)=\theta_{1}^{\underset{‾}{u}}e^{-\lambda_{d}(t-t_{6})}$.

We can deduce the necessary and sufficient condition for the effectiveness of the drug by expressing the inequality *x*_1_((*k*+1)*τ*) < *x*_1_(*kτ*) in terms of dosing period *τ* and unit dose. In the 2-gene case, no explicit closed-form expression can be deduced for the solutions in stages (b) and (f) and numerical methods have to be applied. However, through such analysis, it is observed that the same methodology for analyzing drug effect can be extended to GRNs with multiple interactive genes, although the mathematics involved will become more complicated and sometimes numerical methods must be applied when there is no closed-form solution.

### Simulation results and analysis

**Figure 14:**
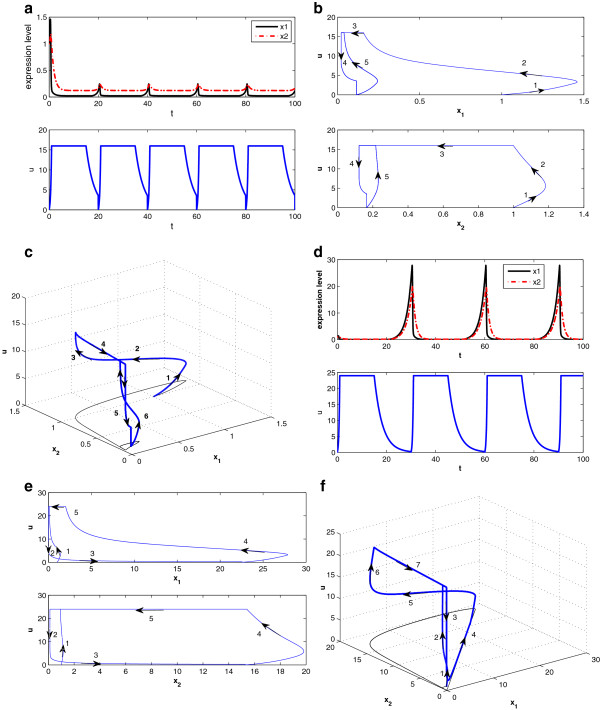
**Drug response follows two different schedules but same drug intake (α = 0.8): (a–c) the state response, state space trajectory, and 3D state-space trajectory at τ = 20, respectively; (d–f) the state response, state space trajectory, and 3D state space trajectory at τ = 30, respectively.** Other parameters are $q_{1}^{u}=2,\beta_{1}=0.1,\beta_{2}=0.1,\eta_{1}=1,\eta_{2}=1,\gamma_{1}=0.2,\gamma_{2}=1,\theta_{1}^{\bar{u}}=8,\theta_{1}^{\underset{‾}{u}}=3,x_{1}(0)=1,x_{2}(0)=1,p_{1}=1,p_{2}=15,\lambda_{d}=0.3$.

When drug input is not present, the disease gene expression will grow unbounded owing to the positive feedback loop between the two genes. Here, we study response of the disease gene expression to drug input and compare two different schedules for *τ* = 20 and *τ* = 30, keeping *α* = 0.8 fixed. The response and state trajectories in 2D and 3D are given in Figure 14a–f, with the first and second rows corresponding to *τ* = 20 and *τ* = 30, respectively. We observe that both cases have periodic responses, but the disease gene expression is much better controlled when *τ* = 20. This is because the drug concentration is high enough in both cases compared to the threshold ($\theta_{1}^{\bar{u}}$), while the decay of the drug concentration is shorter in the case when *τ* = 20. In Figure 14c,f, the 3D state (disease gene expression) trajectories show that the trajectory settles to an inner circle when *τ* = 20, whereas the trajectory settles to an outer circle when *τ*=30. Similar observations apply to Figure 14b,e. Note the scale of *x*-axis of Figure 14e is 20 times bigger than that of Figure 14b.

## Extension and discussion

In previous sections, we have considered the drug-effect on one-gene and a 2-gene case. In this section, we will consider the drug-effect on a target gene in a more sophisticated GRN context.

### 3-gene network with multiple feedback loops

**Figure 15:**
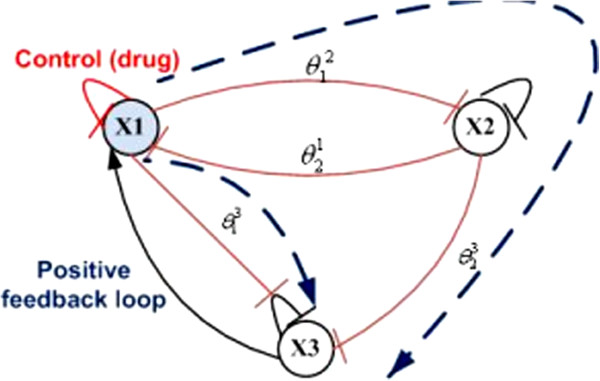
**The 3-gene GRN model.** The solid line is the real interaction between genes. The dashed line is derived to show the positive feedback loop for certain conditions.

Suppose a 3-gene network is given by

(36) $${\dot{x}}_{1}=\beta_{1}s^{-}(x_{2},\theta_{2}^{1})-\gamma_{1}^{u}x_{1}+\eta_{1}x_{3}$$

(37) $${\dot{x}}_{2}=\beta_{2}s^{-}(x_{1},\theta_{1}^{2})-\gamma_{2}x_{2}$$

(38) $${\dot{x}}_{3}=\beta_{3}s^{-}(x_{2},\theta_{2}^{3})-\gamma_{3}s^{-}(x_{1},\theta_{1}^{3})x_{3},$$

where *η*_1_ is a perturbation parameter (from *x*_3_ to *x*_1_), *β*_1_, *β*_2_, *β*_3_ are activation factors, *γ*_2_, *γ*_3_ are degradation factors, and $\theta_{1}^{2},\;\theta_{2}^{1},\;\theta_{1}^{3},\;\theta_{2}^{3}$ are threshold values. $\gamma_{1}^{u}$ is a drug-effect factor. We assume periodic drug intake and drug concentration level follows exponential decay during each period, i.e., $u_{i}(t)=\zeta_{i}e^{-\lambda_{d}(t-k\tau_{i})}$, where *kτ*_*i*_ ≤ *t* ≤ (*k*+1)*τ*_*i*_. A graphical model of the 3-gene network is given in Figure 15. There exist two positive feedback loops between *x*_1_ and *x*_3_.

**Figure 16:**
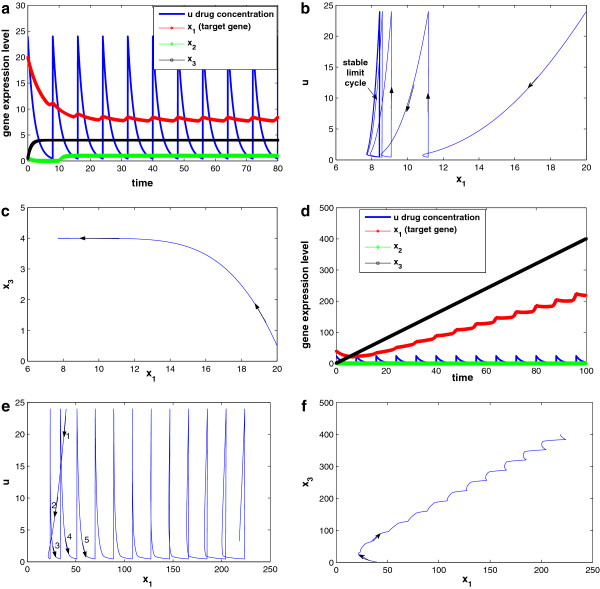
**Simulation results for the 3-gene network under drug perturbation with different initial conditions: (a–c) with initial condition (*x***_***1***_**(*0*)* = 20*), (d–f) with initial condition (*x***_***1***_**(*0*)* = 40*).** Other parameter settings are: $x_{2}(0)=0.7,\;x_{3}(0)=0.5,\;\tau =8,\;u(\mathrm{k\tau})=24,\;q_{1}^{u}=0.21,\beta_{1}=\beta_{2}=1,\;\beta_{3}=4,\;\gamma_{2}=1,\;\gamma_{3}=1,\;\theta_{1}^{2}=10,\;\theta_{2}^{1}=2,\;\theta_{1}^{3}=21,\;\theta_{2}^{3}=10,\;\eta_{1}=0.1,\;\lambda_{d}=0.5$.

When the target gene is in GRN context, not only its expression level is related to drug perturbation, but also depends on network contexts. Several interesting phenomena are observed through our simulations study: 

1. Drug response is related to disease stage. Simulations are performed with different initial target gene expression level (*x*_1_(0)). Figure 16a–c shows the system responses with *x*_1_(0) = 20, which is not too high (corresponding to early disease state). As shown in Figure 16a, *x*_1_ expression level reduces to the range [7.7, 8.4] under periodic drug intake, while *x*_2_ and *x*_3_, the two other interactive genes settle at 1.0 and 4.0, respectively. The system reaches a new steady state (a semi-stable limit cycle, to be exact), with $x_{1}^{s}=\frac{\beta_{1}+\eta_{1}x_{3}^{s}}{\gamma_{1}^{u}}=\frac{\beta_{1}+\eta_{1}\beta_{3}/\gamma_{3}}{\gamma_{1}^{u}}$, $x_{2}^{s}=\frac{\beta_{2}}{\gamma_{2}}$, and $x_{3}^{s}=\frac{\beta_{3}}{\gamma_{3}}$, where *x*_1_ is well controlled. The trajectories of *x*_1_ vs. *u* and *x*_1_ versus *x*_3_ are given in Figure 16b,c, respectively. The semi-stable limit cycle is shown in Figure 16b.

System responses with *x*_1_(0) = 40 (corresponding to late disease state) are shown in Figure 16d–f for comparison. Although the other parameter settings are exactly the same, the drug will not repress the disease gene *x*_1_ (Figure 16d) owing to the interaction between the disease gene *x*_1_ and gene *x*_3_. When $x_{1}(0)=20<\theta_{1}^{3}=21$, Equation (36) becomes ${\dot{x}}_{3}=\beta_{3}s^{-}(x_{2},\theta_{2}^{3})-\gamma_{3}x_{3}$, and thus *x*_3_ is negative regulated by *x*_1_ and converge to $x_{3}^{s}=\frac{\beta_{3}}{\gamma_{3}}$. However, when initial condition $x_{1}(0)=40>\theta_{1}^{3}=21$, Equation (36) becomes ${\dot{x}}_{3}=\beta_{3}s^{-}(x_{2},\theta_{2}^{3})$, and thus *x*_3_ is positively regulated by *x*_2_ and its expression level will keep increasing. As a result, *x*_1_ will keep increasing as well, and a positive feedback loop is formed between *x*_1_ and *x*_3_. This is confirmed by the trajectories of *x*_1_ versus *u* and *x*_1_ versus *x*_3_ given in Figure 16e,f, respectively.

**Figure 17:**
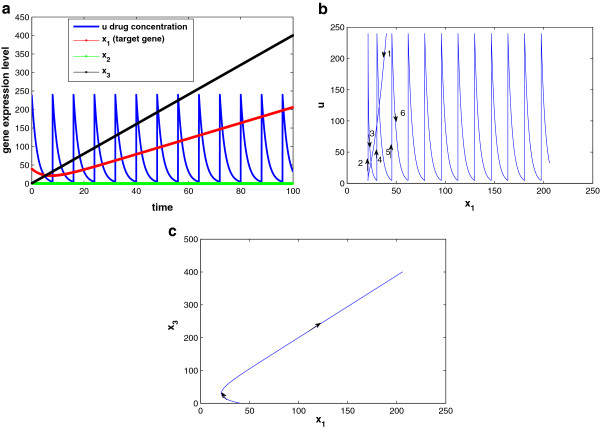
**Simulation results for the 3-gene network under drug perturbation (high dosage), parameter settings are the same with Figure **16**d–f except dosage *u*(*kτ*)* = 240*.**

2. Under certain conditions, single drug perturbation may not be enough. A drug is usually designed to a specific target. In this example, the drug tries to provide negative feedback to the regulation of *x*_1_ (tries to repress *x*_1_); however, since the target gene is interactive (or, in a more general setting, pathways have crosstalk), only repressing the target gene (or blocking the signal of one pathway) may not prevent the target gene from expressing itself through interactions with other genes (or through inter-connected pathways). In our case, *x*_1_ is interactive with *x*_3_. To continue with previous simulation (results shown in Figure 16d–f, we try to increase the drug dosage tenfold from *u*(*kτ*) = 24 to *u*(*kτ*) = 240 with the same dosing period *τ* = 8 trying to bring down the expression level of *x*_1_. However, from system responses shown in Figure 17a–c, it is observed that the drug is not effective although the dosage is increased tenfold.

**Figure 18:**
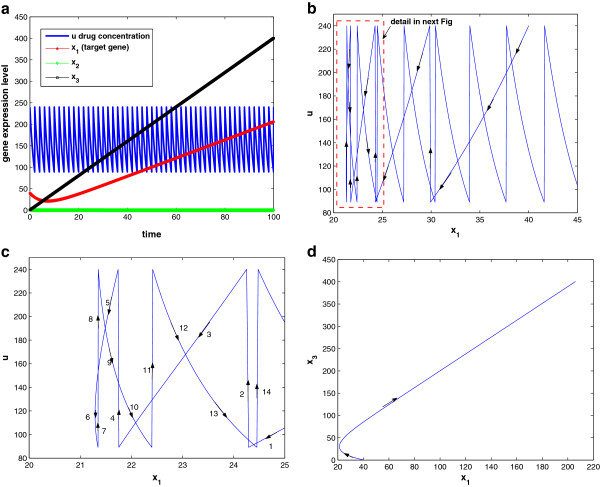
**Simulation results for the 3-gene network under drug perturbation (high dosage and short interval): (a-d) state response, trajectory of *x***_***1 ***_**versus *u*, detailed initial trajectory of *x***_***1 ***_**versus *u*, and trajectory of *x***_***1 ***_**versus*x***_***3***_**, respectively.** Parameter settings are the same with Figure 16 except *u*(*kτ*) = 240 and *τ* = 2.

One step further, not only we increase dosage to *u*(*kτ*) = 240, but also to increase the dosing frequency (dosing period is decreased from *τ* = 8 to *τ* = 2), systems responses are shown in Figure 18a–d, where Figure 18c shows the left part of the trajectory shown in Figure 18b. It can be observed that although the drug perturbation is very strong, and the drug concentration is always staying in domain *D*_5_, drug is still not effective.

From the nonlinear dynamical system perspective, the equation $x_{1}^{s}=\frac{\beta_{1}+\eta_{1}x_{3}^{s}}{\gamma_{1}^{u}}=\frac{\beta_{1}+\eta_{1}\beta_{3}/\gamma_{3}}{\gamma_{1}^{u}}$ represents a semi-stable limit cycle. If the initial condition is from the inside of the limit cycle, then the system will converge to the limit cycle; however, if the initial condition is from the outside of the limit cycle, then the system will diverge from the limit cycle. Such simulation results demonstrate the heterogeneity of the drug’s responses due to the nonlinearities in complex systems, where multiple inputs affect each output and the underpinning structure may include parallel, redundant, and feedback loop processes, it is likely that some cases will not respond to a single drug perturbation no matter how strong it is. As a result, innovative perturbation methods, such as finding a better target or combinatorial therapy, are necessary.

### Simulation of effects of different drugs and a drug combination on *NF*−*κB* pathway

In this article, the models and examples are selected such that they are mathematically tractable and important insights can be obtained, and we can verify the theoretical results with simulation results. For large-scale networks and multiple drugs/drug targets, the proposed model is still applicable; however, analytical results may not be attainable even for this simplistic model. In that case, simulations can be carried out case-by-case. To illustrate this point of view, we carried out a simulation study of the *NF*−*κB* pathway under two different drugs and each drug with different drug targets.

**Figure 19:**
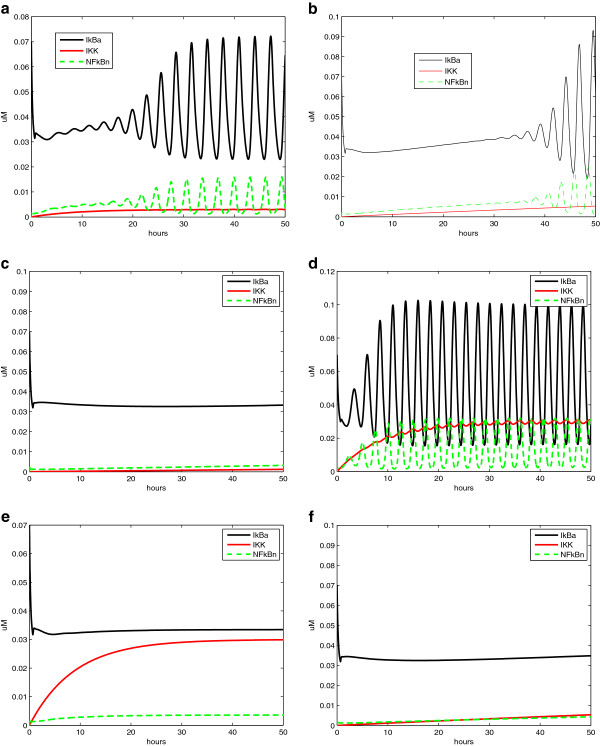
**Simulation results for the *NF−κB * pathway under various drug perturbations with different drug administration. (a)** Effect of continuous stimulus, no drug input. **(b)** Effect of drug X (0.2 *μ*M). **(c)** Effect of drug X (1.0 *μ*M). **(d)** Effect of Bortezomib (65% inhibition). **(e)** Effect of Bortezomib (95% inhibition). **(f)** Effect of combined Bortezomib (65% inhibition) and drug X (0.2 *μ*M). The detailed parameters are available in the Appendix.

*NF*−*κB* signaling regulates inflammation, cell proliferation, and apoptosis by increasing the expression of specific cellular genes in response to a variety of extracellular ligands. How to explore therapeutic strategies to prevent the prolonged activation of the *NF*−*κB* pathway attracts lots of attention [32,33]. An ODE model of the *NF*−*κB* pathway [34] is adopted and the two drugs under consideration are drug X (drug A in [35]) and FDA approved drug proteasome inhibitor Bortezomib (Velcade) [36]. The detailed simulation setup is available in the Appendix and the SIMULINK model is given in Figure 19. The specificity of some drugs to inhibit several of these components of the *NF*−*κB* pathway is one of the concerns. For example, the proteasome which is responsible for the *IκBα* degradation has many other important functions. Thus, Bortezomib modulates a variety of cellular processes that may contribute to toxicity if the dosage is too high [36]. Hence, we design combination therapy to induce a better effect and at the same time to contain toxicity to a certain threshold.

**Figure 20:**
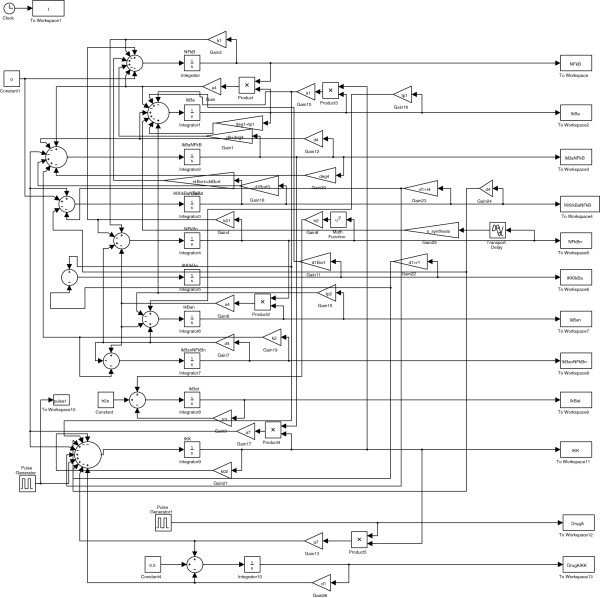
**SIMULINK model for the *NF−κB *pathway.**

To achieve this, drug X is a protein kinase inhibitor, which competitively inhibit IKK with the binding kinetics the same as that of the natural reaction involving *NF*−*κB*:*IκBα* and IKK [35]. While Bortezomib is a proteasome inhibitor that will inhibit *IκBα* degradation, its effect is adjusted through the parameter setting related to individual terms for *IκBα* and *NF*−*κB*:*IκBα* molecules rescued from inhibition of *IκBα* degradation [35]. We first validate the results in [34,35]. In Figure 20a, we show that oscillatory behaviors occur for *NF*−*κB* pathway with constant stimulus. Under this constant stimulus, it is observed in Figure 20b,c that only very high dose of drug X can effectively block *NF*−*κB* nuclear translocation. Similar observation is obtained for Bortezomib from Figure 20d,e, where low drug dosage (65% inhibition) is not effective, while the side effects are unacceptable when the drug is effective (95% inhibition). All the above simulation results are consistent with those in [34,35]. In this article, we go a step further and consider the combination of these two drugs. It is interesting to see in Figure 20f that some combinations of the drugs with non-overlapping toxicities, e.g., combined Bortezomib (65% inhibition) and drug X (0.2 *μ*M), might provide enormous benefit by keeping the level of nuclear *NF*−*κB* low while having tolerable toxicities.

## Conclusions and future work

This article provides systematic mathematical analysis and dynamical modeling of drug effect in the GRN context, where a drug functions as a control input to reduce the elevated target gene expression level. A hybrid systems model is proposed to study the dynamics of the underlying regulatory network under drug perturbation. Drug pharmacology information is incorporated into drug therapeutic response modeling to demonstrate the significant difference in drug effect for different dosing regimens. Considering the complicated nature of gene regulation, this study is a small step towards quantitative modeling of therapeutic effect. We have kept the examples mathematically tractable so that valuable insights and reasonable predictions can be obtained from theoretical analysis.

Compared to our previous work [22], where drug effect was only studied for a specific PK profile (drug concentration only has exponential decay stage) when the drug is targeted to a single gene, three major extensions are provided in this article: (i) we provide analytical results of drug effect under a very general PK profile, where three stages of drug concentration change (increase, equilibrium, and decrease) are considered; (ii) the proposed methodology is applied to interactive genes in a GRN context, with detailed analytical derivations for both one-gene and two-gene cases; and (iii) we perform extensive simulations for a more complicated GRN setting and explain several interesting observations due to multiple feedback loops and the existence of limit cycles.

It is expected that the theoretical framework proposed in this article, when correlated to real biological networks, can help improve drug development productivity and make drug discovery more systematic. During such process, cross disciplinary effort is indispensable. For example, application of such a framework will require experiments designed to elucidate model parameters, such as protein concentration levels and synthesis and degradation speeds. While some parameters may be relatively easy to obtain, others may be difficult to get based on current techniques and model simplification may be necessary; nonetheless, the basic hybrid systems model and the conclusions drawn from it, such as the nature of DERs and the role of limit cycles, will remain valid, only their particular forms being changed to represent experimental instantiation of the model.

## Appendix

See Tables 1 and 2.

**Table 1:** Definition of variables

	
*x*_1_	*NF* − *κB*
*x*_2_	*IκBα*
*x*_3_	*IκBα* : *NF* − *κB*
*x*_4_	*NF* − *κB*_*n*_
*x*_5_	*IκBα*_*n*_
*x*_6_	*IκBα*_*n*_:*NF* − *κB*_*n*_
*x*_7_	*IKK*
*x*_8_	*IKK*:*IκBα*
*x*_9_	*IKK*:*IκBα*:*NF* − *κB*

**Table 2:** Parameter values

**Parameter**	**Reaction type**	**Biochemical reaction**	**Value**	**Unit**
*a*_4_	Complex formation	*NF* − *κB* + *IκBα* → *NF* − *κB* : *IκBα*	30	*μ*M^−1^min^−1^
*a*_7_	Complex formation	*NF* − *κB* : *IκBα* + *IKK* → *NF* − *κB* : *IκBα* : *IKK*	11.1	*μ*M^−1^min^−1^
*a*_1_	Complex formation	*IκBα* + *IKK* → *IκBα* : *IKK*	1.38	*μ*M^−1^min^−1^
*d*_4_	Dissociation	*NF* − *κB* + *IκBα* ← *NF* − *κB* : *IκBα*	0.03	min^-1^
*D*_1_	Dissociation	*NF* − *κB* : *IκBα* + *IKK* ← *NF* − *κB* : *IκBα* : *IKK*	0.075	min^-1^
*D*_1_	Dissociation	*IκBα* + *IKK* ← *IκBα* : *IKK*	0.075	min^-1^
*deg*_1_	Degradation	*IκBα* → 0	0.006	min^-1^
*deg*_4_	Degradation	*NF* − *κB* : *IκBα* → *NF* − *κB*	0.0013	min^-1^
*k*_01_	Transport	*NF* − *κBn* → *NF* − *κB*	0.0048	min^-1^
*tp*_2_	Transport	*IκBαn* → *IκBα*	0.025	min^-1^
*k*_2_	Transport	*NF* − *κBn* : *IκBαn* → *NF* − *κB* : *IκBα*	0.84	min^-1^
*k*_1_	Transport	*NF* − *κB* → *NF* − *κBn*	5.4	min^-1^
*tp*_1_	Transport	*IκBα* → *IκBαn*	0.05	min^-1^
*τ*	Synthesis (delay)	*NF* − *κBn* → *NF* − *κBn* + *IκBα*	40	min
*k*_02_	Inactivation	*IKK* → 0	0.002	min^-1^
*r*_4_+*d*_4_	Catalyzed degradation	*NF* − *κB* : *IκBα* : *IKK* → *NF* − *κB* + *IKK*	11.1	min^-1^
*r*_1_	Catalyzed degradation	*IκBα* : *IKK* → *IKK*	2.22	min^-1^
*s*_synthesis_	Synthesis	*NF* − *κBn* → *NF* − *κBn* + *IκBα*	0.24	min^-1^

### ODE model of the *NF* − *κB* pathway

The ODE model of the *NF* − *κB* pathway is adopted from [34,35].

(39) $$\begin{array}{ccc} \;\frac{dx_{1}}{\text{dt}} & \;= & -a_{4}x_{1}x_{2}+d_{4}x_{3}-a_{4}x_{1}x_{8}+(r_{4}+d_{4})x_{9}\\ & & +\text{de}g_{4}x_{3}-k_{1}x_{1}+k_{01}x_{4} \end{array}$$

(40) $$\begin{array}{ccc} \;\frac{dx_{2}}{\text{dt}} & \;= & -a_{1}x_{2}x_{7}+d_{1}x_{8}-a_{4}x_{1}x_{2}+d_{4}x_{3}-\text{de}g_{1}x_{2}\\ & & -tp_{1}x_{2}+tp_{2}x_{5}+s_{\text{synthesis}}x_{4}(t-\tau ) \end{array}$$

(41) $$\begin{array}{ccc} \;\frac{dx_{3}}{\text{dt}} & \;= & a_{4}x_{1}x_{2}-d_{4}x_{3}-a_{7}x_{3}x_{7}+d_{1}x_{9}\\ & & +k_{2}x_{6}-\text{de}g_{4}x_{3} \end{array}$$

(42) $$\frac{dx_{4}}{\text{dt}}=k_{1}x_{1}-a_{4}x_{4}x_{5}+d_{4}x_{6}-k_{01}x_{4}$$

(43) $$\frac{dx_{5}}{\text{dt}}=tp_{1}x_{2}-tp_{2}x_{5}-a_{4}x_{4}x_{5}+d_{4}x_{6}$$

(44) $$\frac{dx_{6}}{\text{dt}}=a_{4}x_{4}x_{5}-d_{4}x_{6}-k_{2}x_{6}$$

(45) $$\begin{array}{l} \frac{dx_{7}}{\text{dt}}=k(t)-k_{02}x_{7}-a_{1}x_{2}x_{7}+(d_{1}+r_{1})x_{8}\\ \;-a_{7}x_{3}x_{7}+(d_{1}+r_{4})x_{9} \end{array}$$

(46) $$\frac{dx_{8}}{\text{dt}}=a_{1}x_{2}x_{7}-(d_{1}+r_{1})x_{8}$$

(47) $$\frac{dx_{9}}{\text{dt}}=a_{7}x_{3}x_{7}-(d_{1}+r_{4})x_{9}$$

## Competing interests

The authors declare that they have no competing interests.
